# Validation of a method to partition the base deficit in meningococcal sepsis: a retrospective study

**DOI:** 10.1186/cc3760

**Published:** 2005-07-08

**Authors:** Ellen O'Dell, Shane M Tibby, Andrew Durward, Jo Aspell, Ian A Murdoch

**Affiliations:** 1 Fellow, Department of Paediatric Intensive Care, Guy's and Saint Thomas' Hospitals, London, UK; 2 Consultant, Department of Paediatric Intensive Care, Guy's and Saint Thomas' Hospitals, London, UK; 3 Resident, Department of Paediatric Intensive Care, Guy's and Saint Thomas' Hospitals, London, UK; 4 Consultant, Department of Paediatric Intensive Care, Guy's and Saint Thomas' Hospitals, London, UK

## Abstract

**Introduction:**

The base deficit is a useful tool for quantifying total acid–base derangement, but cannot differentiate between various aetiologies. The Stewart–Fencl equations for strong ions and albumin have recently been abbreviated; we hypothesised that the abbreviated equations could be applied to the base deficit, thus partitioning this parameter into three components (the residual being the contribution from unmeasured anions).

**Methods:**

The two abbreviated equations were applied retrospectively to blood gas and chemistry results in 374 samples from a cohort of 60 children with meningococcal septic shock (mean pH 7.31, mean base deficit -7.4 meq/L). Partitioning required the simultaneous measurement of plasma sodium, chloride, albumin and blood gas analysis.

**Results:**

After partitioning for the effect of chloride and albumin, the residual base deficit was closely associated with unmeasured anions derived from the full Stewart–Fencl equations (*r*^2 ^= 0.83, *y *= 1.99 – 0.87*x*, standard error of the estimate = 2.29 meq/L). Hypoalbuminaemia was a common finding; partitioning revealed that this produced a relatively consistent alkalinising effect on the base deficit (effect +2.9 ± 2.2 meq/L (mean ± SD)). The chloride effect was variable, producing both acidification and alkalinisation in approximately equal proportions (50% and 43%, respectively); furthermore the magnitude of this effect was substantial in some patients (SD ± 5.0 meq/L).

**Conclusion:**

It is now possible to partition the base deficit at the bedside with enough accuracy to permit clinical use. This provides valuable information on the aetiology of acid–base disturbance when applied to a cohort of children with meningococcal sepsis.

## Introduction

Metabolic acidosis is a common biochemical finding in critically ill patients [1]. The prognostic significance of this entity is recognised in many mortality risk scores, in which the predicted risk increases in proportion to the degree of acidosis [2-4]. The commonest bedside tool for quantifying a metabolic acidosis is the base deficit [5]. Although the base deficit is an accurate measure of total acute acid–base derangement, it cannot delineate the various aetiologies that can contribute to an acidosis [6,7]. Broadly speaking, these include tissue acids (which dissociate into lactate and other, unmeasured anions), hyperchloraemia (a 'normal anion gap' acidosis), and weak acids (traditionally known as buffers, of which albumin is the most important). It is not uncommon for the three aetiologies to coexist in the critically ill patient; furthermore, the relative contribution from each can vary with time [8,9]. The cause, treatment, and perhaps prognostic significance of each of these aetiologies differ; a tool to partition the base deficit for each component would therefore be useful [10].

Recent insights into acid–base physiology (the Stewart–Fencl approach) have provided a method of quantifying each component of the acid–base status [6,7,11]. However, the necessary physicochemical calculations are cumbersome and require the simultaneous measurement of many biochemical variables. Two abbreviated versions of the Stewart–Fencl equations have recently been derived: one for albumin, the other for chloride [12,13]. We hypothesised that, by applying these to the base deficit, the residual would reflect the acidifying effect of unmeasured anions, thus partitioning the base deficit into its three components. Our secondary hypothesis was that the loss of accuracy as a consequence of applying these abbreviated formulae to the base deficit would not be great enough to compromise clinical validity. We have investigated this retrospectively in a cohort of 60 children with meningococcal septic shock. This patient group was chosen for two reasons: metabolic acidosis is a common occurrence in itself, and so are derangements in all three components contributing to the acidosis.

## Methods

The study was approved by the Institutional Ethics Committee, which waived the need for informed consent.

### Patients

We examined data retrospectively from 68 consecutive patients with meningococcal sepsis admitted to the paediatric intensive care unit from January 2001 to June 2003. Cases were identified from the departmental database. Patients with meningococcal meningitis without septic shock were excluded. Septic shock was defined as the need for more than 40 ml/kg of fluid resuscitation within 4 hours of presentation to hospital or the requirement for inotropic medication [14]. All blood samples taken during the first 72 hours of admission, in which a full chemistry profile was measured simultaneously with arterial blood gas analysis, were analysed.

After exclusion of those without septic shock, full data were available for 374 blood samples from 60 patients (giving a median of six samples per patient). Patient demographics were as follows: median (interquartile) age 2 years (0.8 to 9.5), weight 13 kg (10 to 19), Paediatric Index of Mortality version 2 (PIM2)-derived mortality risk 11.0% (6 to 16), crude mortality 10.0% (PIM2-predicted death rate 13.8%). In addition, 88% of patients required mechanical ventilation, 82% received inotropes, and the amount of fluid administered in the first 24 hours after admission was 158 ± 65 ml/kg (mean ± SD).

### Blood chemistry analysis

Arterial blood gases and chemistry were measured with the Instrumentation Laboratory 1640 blood gas analyser (Lexington, MA, USA) and Synchron LX20 (Beckman Coulter Inc., High Wycombe, Buckinghamshire, UK) The total base deficit (BD_tot_) was calculated according to the algorithm for 'standard base deficit' in the blood gas analyser, which necessitated the concurrent measurement of haemoglobin. Plasma albumin was measured by binding of bromocresol green dye, and whole blood lactate by the enzymatic method (YSI 2300 STAT plus analyser; Yellow Springs Instruments, Yellow Springs, OH, USA). The precision values for the above analysers were as follows: pH 0.009 to 0.005 (at pH values of 7.15 and 7.66 respectively), p_CO2 _2.74 to 2.78%, ion-specific electrodes all less than 2%, albumin 6.2% and 3.0% (at albumin concentrations of 13 and 37 g/L, respectively), and lactate 2.0%.

### Formulae to partition the base deficit

BD_tot _is influenced by three factors: weak acids, of which albumin is dominant (BD_alb_); strong ions, of which chloride concentration (relative to sodium) is the most important (BD_cl_); and net unmeasured anions from tissue acids (BD_UMA_). Blood lactate can be considered as either a strong ion (if measured) or an unmeasured anion (if unmeasured). For the purposes of this study we designated lactate as an unmeasured anion.

These three factors can exert an acidifying (hyperalbuminaemia, hyperchloraemia, excess unmeasured anions) or an alkalinising (hypoalbuminaemia, hypochloraemia, excess unmeasured cations) effect on the total base deficit, according to their net charge [6,7,11].

If formulae quantifying the effect of albumin and chloride on the base deficit are applied and subtracted from the total base deficit, the remainder should equal the contribution from net unmeasured anions (or cations if the residual base deficit is positive), so that

BD_tot _- BD_alb _- BD_Cl _= BD_UMA_

If this method is accurate, the residual (BD_UMA_) should therefore approximate unmeasured anions calculated from the Stewart–Fencl equations. After considering the precision of the blood gas and chemistry analysers, the expected loss of accuracy due to the abbreviated nature of the base deficit equations, and the normal value for the Stewart–Fencl strong ion gap (see below), we set an *a priori *limit of 3 meq/L for the standard error of the estimate (SEE).

The formulae for base deficit used in this study have been derived elsewhere [12,13], and are as follows:

BD_alb _= [42 - albumin (g/L)] × 0.25

BD_Cl _= [Na^+^] - [Cl^-^] - 32

A full explanation of the Stewart–Fencl methodology is reported elsewhere [6,7,11,15]; however, a brief explanation is included in Additional file 1. The equations are as follows:

Unmeasured anions (strong ion gap) = strong ion difference (measured) - strong ion difference (effective)

Strong ion difference (measured) = [Na^+ ^+ K^+ ^+ Ca^2+ ^+ Mg^2+^] - [Cl^- ^] (all in meq/L)

Strong ion difference (effective) = (12.2 × p_CO2_/(10^-pH^)) + 10 × [Alb] (g/L) × (0.123 × pH - 0.631) + [PO_4_^2-^] (mmol/L) × (0.309 × pH - 0.469).

### Statistical analysis

Data are reported as means and SD. Agreement between unmeasured anions calculated from the base deficit (BD_UMA_) and Stewart–Fencl methods (strong ion gap) was assessed by linear regression with the use of the ordinary least-squares method (Microsoft Excel).

## Results

**Figure 1:**
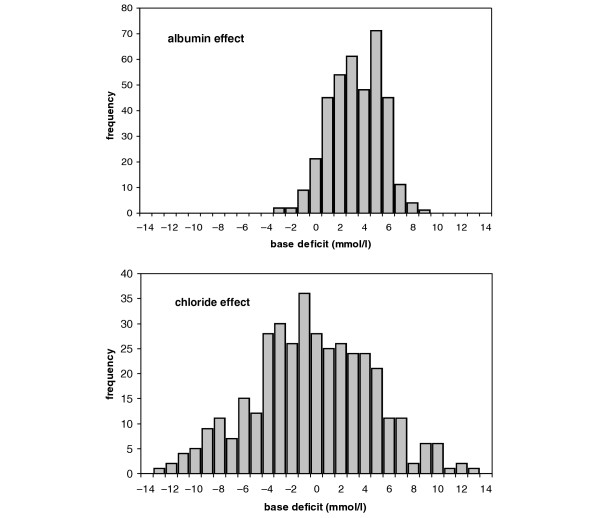
Histograms demonstrating the effect of **(a) **albumin and **(b) **chloride on total base deficit for all blood samples (*n *= 374).

Acid–base and biochemical results are shown in Table 1. A significant metabolic acidosis was seen for the group as a whole (mean pH 7.31, BD_tot _-7.4). The unmeasured anion-related base deficit was greater than the total base deficit; this was predominantly due to the alkalinising effect of hypoalbuminaemia (mean albumin effect on base deficit +2.9; Table 1). This is also shown in the histogram for BD_alb _(Fig. 1a), showing an alkalinising effect in 91% of samples. The influence of chloride was variable, producing both acidifying and alkalinising effects in approximately equal proportions (50% and 43%, respectively; Fig. 1b). It is also notable that the range of chloride effect on base deficit was more extreme than that for albumin (SD 5.0 versus 2.2; Table 1 and Fig. 1).

**Figure 2:**
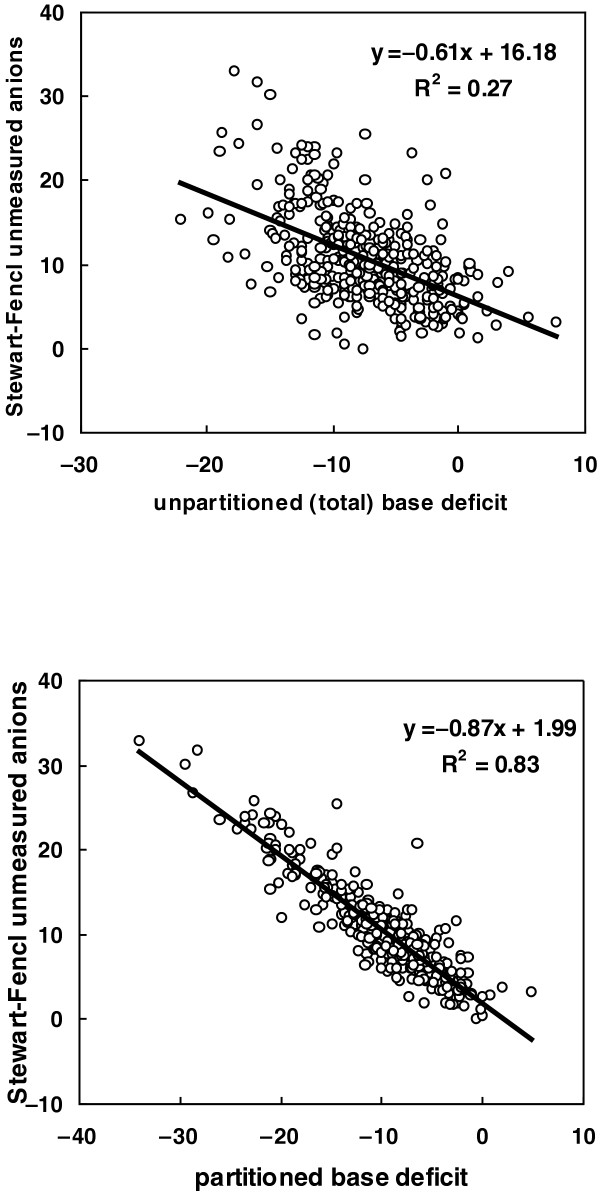
Scatter plots showing relationship between Stewart–Fencl derived unmeasured anions and base deficit. **(a) **unpartitioned (total) base deficit and **(b) **partitioned (unmeasured anion fraction) base deficit.

Not surprisingly, BD_tot _was weakly associated with Stewart–Fencl unmeasured anions (*r*^2 ^= 0.27; Fig. 2a). However, after adjustment for chloride and albumin, BD_UMA _showed a strong, linear association with Stewart–Fencl unmeasured anions (*r*^2 ^= 0.83; Fig. 2b). The full regression equation was Stewart–Fencl-derived UMA = 1.99 - (0.87 × BD_UMA_), SEE 2.29 meq/L.

Finally, it is noted that the regression analysis used multiple samples taken from each patient; thus each measurement cannot be considered truly independent in a statistical sense (even though an individual patient's base deficit may have changed markedly over the 72 hours after admission). In this setting, multiple measurements taken on an atypical patient could potentially bias the regression analysis. To investigate this we reanalysed the data in two ways. First, a standardised residual plot was inspected, which did not reveal obvious deviation from normality among the residuals, nor any extreme outliers. Second, we repeated the regression analysis, using one measurement only per patient (*n *= 60). Measurements were chosen by means of the random number generator in Excel using a uniform distribution, whereby the sample with the largest assigned random number from each patient's group of samples was chosen. This produced remarkably similar results: *r*^2 ^= 0.854, Stewart–Fencl-derived UMA = 2.39 - (0.871 × BD_UMA_), SEE 2.17 meq/L. We therefore conclude that the above approach is valid.

## Discussion

These findings demonstrate that the base deficit can be partitioned at the bedside by the application of two simple formulae, requiring the measurement of plasma sodium, chloride and albumin concurrently with the arterial blood gas. This was validated by comparing the unmeasured anion portion of the base deficit with that calculated from the Stewart–Fencl equations, yielding a high coefficient of determination (*r*^2 ^= 0.83). However, to assess whether this model is accurate enough for clinical use, we must consider three other aspects of the regression analysis, namely the SEE (2.29 meq/L), the slope (-0.87) and the intercept (1.99 meq/L).

Inspection of the residual plots (data not shown) did not reveal an unusual pattern; furthermore, the residuals seemed normally distributed with consistent variance. Thus we can say that about 95% of the time the true unmeasured anions will lie within ± 4.5 meq/L of that estimated by the partitioned base deficit (1.96 × standard error).

The sources of this error are threefold, including both the abbreviated albumin and chloride equations and the fact that phosphate, the other major weak acid, is not accounted for. Albumin charge is a function of pH [7,11,16,17]; the albumin error will therefore increase with the degree of acidosis (both respiratory and metabolic).

However, this effect is not large; Story has estimated a typical error to be less than ± 1 meq/L [12], which is confirmed in the present study (data not shown). The abbreviated chloride equation does not account for the effect of variation in other cations (including potassium, calcium and magnesium). Lastly, omitting phosphate from the base deficit equations results in an error in unmeasured anion estimation of 1.8 meq/L for every 1 mmol/L change in phosphate concentration (again, this will alter slightly depending upon pH).

The slope of the regression equation (-0.87) can be interpreted as meaning that a decrease in base deficit of -10 meql/L will represent an increase of 8.7 meq/L in unmeasured anions; in essence this is close enough to be considered as an inverse equimolar relationship. It is also notable that the intercept, which occurs when the base deficit is equal to zero, yields an estimated unmeasured anion value of 2 meq/L, which is consistent with the normal value for this parameter [17].

In summary, we feel that the properties described above permit bedside partitioning of the base deficit, provided that the user is aware of the limitations and sources of error. Partitioning provides the clinician with valuable information about the aetiology of an acidosis, which can have implications for treatment and prognosis. Examples are outlined below.

Underestimation of tissue acidosis: critically ill patients have a high incidence of hypoalbuminaemia that produces an alkalinising effect, masking the true degree of 'tissue acidosis' [10,17,18]. In addition, several authors have documented relative hypochloraemia when tissue acidosis occurs, postulating that this represents a compensatory mechanism [8,19]. Neither phenomenon will be apparent from an unpartitioned base deficit.

Recognition of iatrogenic causes of an acidosis: the use of albumin solutions for resuscitation is common in paediatrics, and may become more widespread in adult practice since the publication of recent safety data [20]. Albumin-based fluids can propagate an acidosis by two mechanisms: they increase plasma albumin concentration, and most contain an abundant source of chloride. The latter mechanism is common to any fluid containing a high concentration of chloride relative to sodium (for example 0.9% saline) [21-25]. Persistent acidosis in this setting might be interpreted erroneously as being due to tissue hypoperfusion from inadequate resuscitation, provoking an unnecessary escalation of therapy.

Prognosis: the prognostic value of an acidosis related to unmeasured anions is uncertain, with studies producing conflicting results [26-30]; this may be due to the variable aetiology and composition of unmeasured anions (such as ketones, organic acids, sulphate and acetate). Conversely, several studies have suggested that a hyperchloraemic acidosis may carry a more favourable prognosis [27,28,31].

A potential criticism of the partitioning approach is that it may offer the same information as the anion gap. This is partly true, provided that the anion gap is corrected for albumin [17,18]. However, the anion gap alone cannot diagnose a mixed acidosis (unmeasured anion plus hyperchloraemic), which occurs frequently in critically ill patients. By partitioning the base deficit, we are in effect combining these two parameters into a single measurement that contains both quantitative and qualitative information.

This study did not attempt to address the role of lactate, but merely sought to validate a method for partitioning the base deficit. The prognostic and therapeutic value of lactate measurement is well established [27,32,33]; this anion is routinely measured as a point-of-care test in many critical care units. We suggest that lactate measurement is complementary to the partitioned base deficit approach, providing a method of further subdividing BD_UMA _into lactate and non-lactate components. This is important, because the two components are not tightly correlated [8,9].

## Conclusion

In summary, we have validated two simple equations that permit partitioning of the base deficit into three components (chloride, albumin and unmeasured anions), providing for a more detailed bedside analysis of acid–base disturbances. We have found this to be useful in everyday clinical practice.

## Key messages

• 	It is possible, by application of two simple equations, to partition the base deficit into three components: chloride, albumin and unmeasured anions.

• 	This requires simultaneous measurement of an arterial blood gas, and venous plasma sodium, chloride and albumin.

• 	Agreement between unmeasured anions calculated from the partitioned base deficit and from the full Stewart–Fencl equations produces good agreement (*r*^2 ^= 0.83) in a cohort of patients with meningococcal sepsis.

• 	The partitioned base deficit reveals a predominantly alkalinising effect of albumin in this group (effect +2.9 ± 2.2 meq/L (mean ± SD)).

• 	The effect of chloride on the base deficit was more variable, producing significant acidifying and alkalinising effects in almost equal measure (effect -0.5 ± 5.0 meq/L (mean ± SD)).

## Abbreviations

BD_alb _= base deficit due to albumin; BD_Cl _= base deficit due to chloride; BD_tot _= total base deficit; BD_UMA _= base deficit due to unmeasured anions; PIM2 = Paediatric Index of Mortality version 2; SEE = standard error of the estimate.

BD_alb _= base deficit due to albumin; BD_Cl _= base deficit due to chloride; BD_tot _= total base deficit; BD_UMA _= base deficit due to unmeasured anions; PIM2 = Paediatric Index of Mortality version 2; SEE = standard error of the estimate.

## Competing interests

The author(s) declare that they have no competing interests.

## Authors' contributions

EOD performed data collection, preliminary data analysis and co-wrote the first draft of the manuscript. SMT conceived the study, performed data analysis and co-wrote the first draft of the manuscript. AD participated in the design of the study, derived one of the base deficit formulae and advised on data analysis. JA performed data collection. IAM supervised the project and participated in study design. All authors read and approved the final manuscript.

## Supplementary Material

Additional File 1 A Word document describing Stewart's physiochemical approach to acid–base balance. Click here for file

## Figures and Tables

**Table 1:** Acid–base and biochemical parameters for all samples (*n *= 374)

Variable	Unit	Mean	SD
PH	-	7.31	0.09
p_CO2_	Kpa	4.6	1.1
BD_tot_	meq/L	-7.4	4.7
BD_alb_	meq/L	+2.9	2.2
BD_Cl_	meq/L	+0.16	5.0
BD_UMA_	meq/L	-10.0	5.7
Sodium	meq/L	140	5.1
Chloride	meq/L	109	6.9
Albumin	g/L	30.5	8.8
Lactate	meq/L	2.5	2.3
Strong ion gap	meq/L	10.7	5.5

BD_alb_, base deficit due to albumin; BD_Cl_, base deficit due to chloride; BD_tot_, total base deficit; BD_UMA_, base deficit due to unmeasured anions.
