# Supplementary material for: Validation of a method to partition the base deficit in meningococcal sepsis: a retrospective study
Source: Crit Care. 2005 Jul 8;9(4):R464–70. doi: 10.1186/cc3760 (PMC1269470; doi:10.1186/cc3760)
Supplement: Additional File 1 — A Word document describing Stewart's physiochemical approach to acid–base balance. [file cc3760-S1.doc]

**Additional file 1: Stewart’s Physiochemical Approach to Acid Base Balance**

Stewart applied three physicochemical principles to acid-base analysis, namely: the law of mass action, the principle of mass conservation and the preservation of electroneutrality. From this he proposed that three independent variables determine plasma pH by altering the degree to which water dissociates into hydrogen and hydroxyl ions. The three variables are PCO2, total weak acid (ATOT, of which albumin and phosphate are the most important) and the strong ion difference (SID). A strong ion is an element within a molecule that completely dissociates in a solution at physiological pH (e.g. NaCl will completely dissolve in H2O into Na+ and Cl- ions). Thus the measurable SID (also known as SIDa) represents the difference in plasma between the sum of the measured strong cations (Na+, K+, Ca2+, Mg2+) and strong anions (Cl-, lactate). There are, of course other anions and cations within plasma that we do not measure that influence the “true” value of SID. These unmeasured anions make up the strong ion gap (SIG), and in health are present in very small amounts (e.g. ketones, sulphate, anions from organic acids). In certain disease states however, the value of SIG may increase considerably becoming positive in states of excess unmeasured anions, and negative with excess unmeasured cations (a confusing terminology!). SIG can be estimated by the charge difference between SIDa and the charge on ATOT.

These entities influence acid base balance in a predictable manner. Metabolic acidosis results from: a decrease in SIDa (e.g. hyperchloraemia), an excess of unmeasured anions (producing a positive SIG e.g. ketoacidosis), or an increase in weak acid concentration (hyperalbuminaemia). Conversely metabolic alkalosis occurs during the opposite situations (hypochloraemia, unmeasured cations, hypoalbuminaemia).

A major difference between the Stewart approach and the traditional methodology, which centres upon the Henderson-Hasselbalch equation, pertains to the role of bicarbonate. The structure of the Henderson-Hasselbalch equation implies that bicarbonate is an independent variable, i.e. one that can vary independently of PCO2. This assumption is inherent in contemporary acid-base theory (many disease states are referred to as “bicarbonate losing”). The Henderson equation, on which the Henderson-Hasselbalch modification is based, shows clearly that bicarbonate cannot vary independently of carbonic acid (and hence CO2), or hydrogen; all are linked. Stewart recognises this, indeed all the equations that quantify the effect of the independent variables (PCO2, SIG, SIDa, ATOT) and hence measure hydrogen ion concentration can be rearranged to predict bicarbonate with the same accuracy.

Henderson-Hasselbalch equation: pH = pKa + log {[HCO3-] / (PCO2 x 0.03)}

Henderson equation: constant = [H+] x [HCO3-] / (PCO2 x 0.03)
